# YOUNG OVARY FACTORS TRANSFER FEMALE COGNITIVE RESILIENCE TO THE AGED MALE BRAIN

**DOI:** 10.21203/rs.3.rs-10769557/v1

**Published:** 2026-09-03

**Authors:** Hyunbin D. Huh, Yasuhiro Fuseya, Karishma J.B. Pratt, Gregor Bieri, Amber R. Philp, Gabriel Avillion, Ariella Shikanov, Saul A. Villeda

**Affiliations:** 1. Department of Anatomy, University of California San Francisco, San Francisco, CA 94143, USA; 2. Obstetrics & Gynecology, University of Michigan Ann Arbor, Ann Arbor, MI 48109, USA; 3. Department of Physical Therapy and Rehabilitation Science, University of California San Francisco, San Francisco, CA 94143, USA; 4. Bakar Aging Research Institute, University of California San Francisco, San Francisco, CA, 94143, USA

## Abstract

Women typically exhibit slower age-associated cognitive decline than men. Here, we show that young ovary-derived blood factors transfer female cognitive resilience to the aged male brain. Administration of young female-derived blood plasma elicited greater cognitive and neurogenic enhancements in aged male recipient mice compared to young male-derived plasma. Heterochronic young ovary transplantation likewise boosted cognition, neurogenesis and synaptic-related transcriptional signatures in aged males. We identify secreted frizzled-related protein 4 (SFRP4) as an ovary-enriched circulating factor in young female plasma, of which systemic elevation in aged male mice ameliorates cognitive impairments. Mechanistically, we identify bone-derived pro-youthful factor osteocalcin as a downstream peripheral mediator of SFRP4-induced cognitive benefits. Together, these findings highlight an ovary–bone–brain axis through which blood factors confer female cognitive resilience in aging.

Systemic rejuvenating interventions such as exercise, heterochronic parabiosis and young blood plasma administration have been shown to improve age-related cognitive dysfunction^1–8^. Moreover, emerging evidence indicates that circulating factors elevated in blood by different systemic interventions promote rejuvenation through distinct molecular mechanisms^9,10^, raising the possibility of rejuvenating factors that differ across biological contexts, including sex. However, whether circulating factors exert sex-dependent restorative effects on cognition remains unclear. Defining the role of systemic factors in shaping sex-specific brain resilience will be critical for developing therapeutic strategies that harness endogenous protective pathways to rescue cognitive decline in the growing older population.

Women generally live longer than men across populations^11,12^. Additionally, females exhibit slower biological aging – an advantage conserved across mammalian species that underscores the influence of biological sex on aging trajectories^13–15^. Notably, age-related cognitive decline typically progresses more slowly in females than in males, with a memory advantage that persists into old age^16–19^, implicating female-specific biological factors in cognitive resilience against brain aging. Female cognitive resilience has been attributed in part to a higher cognitive reserve established earlier in life^20^, implicating youthful female-specific biological factors in cognitive resilience that counter brain aging. Interestingly, premenopausal oophorectomy is associated with an increased risk of cognitive decline and dementia in women^21–24^, and ovariectomy in young female mice impairs learning and memory^25,26^. In contrast, young ovary transplantation extends lifespan and confers long-term systemic benefits in aged female recipient mice^27–29^. These findings implicate the ovary as a systemic regulator of aging. However, whether blood-borne ovary-derived factors mitigate brain aging and rescue cognitive decline remains to be explored.

Here, we show that young ovary-derived circulating blood factors exert sex-transcending restorative effects on brain aging in males. Blood plasma from young females more robustly ameliorates cognitive and neurogenic impairments in aged male mice than plasma from young males, and heterochronic young ovary transplantation likewise enhances adult neurogenesis and cognition at old age in males. We identify secreted frizzled-related protein 4 (SFRP4) as an ovary-enriched circulating factor elevated in young female plasma and following young ovary transplantation in aged male mice, with increased levels correlating with improved cognitive performance. Systemic administration of SFRP4 rescues cognition in aged male mice through increased levels of the bone-derived pro-youthful blood factor osteocalcin (OCN). This works highlights an ovary–bone–brain axis that underlies female-specific cognitive resilience in aging.

## Circulating factors in young female plasma induce greater hippocampal enhancements than young male plasma in aged male mice

To begin, we investigated whether sex differences in circulating factors in blood plasma derived from young male or young female mice differentially impacted cognitive function in the aged male hippocampus – a brain region governing learning and memory and highly vulnerable to the effects of aging^9^. Aged (22 months) male mice received intravenous administrations of young (2 months) male or young female blood plasma or saline control, delivered eight times over a four-week period. To compare the capacity of young male and young female plasma to reverse age-associated impairments in hippocampus-dependent cognition, animals were subjected to a battery of behavioral paradigms, including Y maze, novel object recognition (NOR), and the radial arm water maze (RAWM) (Fig. 1a). Aged male mice treated with young male or young female plasma, but not saline control, exhibited increased preference for the novel arm (Fig. 1b) and novel object (Fig. 1c) compared to the familiar condition during Y maze and NOR testing, respectively, indicative of improvements in both working memory and object recognition. While all young plasma treatment groups exhibited improved cognitive function, statistical significance relative to saline controls was observed only in aged male mice receiving young female plasma, suggesting a potentially greater cognitive benefit of female-derived plasma (Fig. 1b,c). This difference between young plasma treatments became most pronounced during RAWM testing. In line with previous reports^2,5,8^, young plasma administration improved spatial learning and memory, with aged mice committing less errors relative to saline treated aged animals during the testing phase (Fig. 1d). However, young female plasma treatment resulted in a more robust cognitive enhancement in spatial memory than young male plasma treatment in aged male mice (Fig. 1d). No adverse effects in health metrics were observed across groups (Extended Data Fig.1a–d).

At a cellular level, exposure to young blood has been shown to enhance regenerative capacity in the aged brain^1,4^. Therefore, we next compared the effects of young male and young female plasma on adult neurogenesis in the aged male hippocampus by immunohistochemical analyses. Administration of young female plasma increased the number of newly generated neurons labeled by Doublecortin (Dcx) in the dentate gyrus (DG) region of the aged male hippocampus compared with both young male plasma and saline treated groups (Fig. 1e,f).

To more broadly define cellular and molecular changes elicited in the aged male hippocampus in response to administration of young male versus young female plasma, we performed hippocampal single nucleus RNA-sequencing (snRNAseq) (Fig. 1g). A total of 13 cell clusters were identified (Fig. 1h), and cell-type composition was compared across treatment group (Extended Data Fig.1e,f). Analysis of differentially expressed genes (DEGs) revealed substantial overlap between young plasma treatments, with approximately 75% of DEGs observed in the young male plasma group shared with the young female plasma group, and nearly all transcriptional changes occurring in the same direction (Fig. 1i,j). Both young male and young female plasma treatments induced prominent transcriptional remodeling in neuronal populations across the DG, inhibitory neurons, and CA1 and CA3 regions of the hippocampus relative to the aged saline-treated control group (Fig. 1k,l and Extended Data Fig. 1g,h). Gene ontology (GO) analysis identified enrichment of biological processes related to synaptic plasticity and neuronal projection guidance across young plasma treatment conditions (Fig. 1m–q and Extended Data Fig. 1i–k).

We next examined transcriptional responses preferentially associated with young female plasma administration. DEGs that were selectively altered following young female plasma treatment were predominantly detected in hippocampal neuronal populations across CA1, CA3, and the DG regions (Fig. 1r). GO analysis of female plasma-induced DEGs highlighted homeostatic biological processes, including ribosomal translation, biosynthesis, and intracellular transport (Fig. 1s). Notably, ribosomal genes showed greater transcriptional changes than other gene categories and were broadly upregulated across hippocampal neurons in aged male mice receiving young female plasma compared to young male plasma or saline control (Fig. 1t,u and Extended Data Fig. 1l). These findings are consistent with prior reports demonstrating that brain aging is accompanied by a global decline in homeostatic gene expression, including pathways related to ribosomal function, metabolism, and transport^30^, and suggest a link between young female plasma and restoration of ribosomal gene expression. Together, these behavioral, cellular and transcriptomics data indicate sex dimorphic differences in the circulating factors present in young male versus young female plasma and suggest potential for more robust rejuvenation of the aged male hippocampus through female-specific blood factors.

## Heterochronic young ovary transplantation induces hippocampal improvements in aged male mice

Given the robust beneficial effect of young female plasma on the aged male hippocampus, and the reported protective role of the ovary in age-associated brain dysfunction^21–25^, we next examined whether heterochronic ovary transplantation could rescue age-related cognitive impairments. Aged male mice underwent young ovary transplantation, using an approach previously shown to maintain viable and well-vascularized ovarian grafts^31^, or sham surgery as a control, and hippocampal-dependent cognitive function was assessed using the Y-maze, NOR, and RAWM paradigms (Fig. 2a and Extended Data Fig. 2a). Heterochronic young ovary transplanted aged male mice exhibited a significant preference for the novel arm, but not the novel object, compared to a familiar condition, whereas sham aged male mice displayed no bias in either Y maze or NOR tests (Fig. 2b,c). During the training phase of the RAWM paradigm, learning performance followed similar overall trajectories between groups (Fig. 2d). However, during the testing phase, heterochronic young ovary-transplanted aged male mice demonstrated improved memory for platform location relative to sham controls (Fig. 2d). No adverse effects in health metrics were observed across groups (Extended Data Fig. 2b–e). At a cellular level, we observed increased adult neurogenesis in the hippocampus of aged male mice following heterochronic young ovary transplantation compared to sham control (Fig. 2e,f).

To gain mechanistic insight into the hippocampal cellular and molecular changes induced by young ovary transplantation, we employed snRNAseq. Hippocampi were isolated from aged male mice following heterochronic young ovary transplantation or sham surgery (Fig. 2g). A total of 12 cell clusters were identified (Fig. 2h), and cell-type composition was compared across groups (Extended Data Fig. 2f,g). Heterochronic young ovary transplantation induced pronounced, cell type-specific transcriptional remodeling across neuronal populations, particularly within the DG and CA1 regions of the aged male hippocampus compared with sham controls (Fig. 2i). GO analysis of DEGs revealed enrichment of neurogenesis-related biological processes in the DG (Extended Data Fig. 2j,k) and synaptic plasticity-associated pathways in CA1 (Fig. 2l,m). Both DG and CA1 neuronal populations also showed enrichment of fundamental cellular processes, including ribosomal translation and biosynthetic pathways (Fig. 2j,l), mirroring transcriptional programs observed following young female plasma administration. Notably, ribosomal genes exhibited more pronounced transcriptional changes than other gene categories and were broadly upregulated across hippocampal neurons in the DG and CA1 regions of aged male mice following ovary transplantation compared to sham controls (Extended Data Fig. 2h–k).

We next examined transcriptional programs shared between heterochronic young ovary transplantation and young female plasma administration relative to aged control conditions. snRNAseq datasets from aged male mice undergoing young ovary transplantation or receiving young female plasma were integrated with corresponding control samples (Fig. 2n). A total of 10 cell clusters were identified (Fig. 2o), and cell-type composition was compared across groups (Extended Data Fig. 2l,m). Analysis of conserved DEGs demonstrated strong transcriptional concordance between the two interventions, with approximately 63% of DEGs identified after ovary transplantation also observed following young female plasma treatment and changing in a predominantly unidirectional manner (Fig. 2p,q and Extended Data Fig. 2n). Both heterochronic young ovary transplantation and young female plasma treatment induced pronounced transcriptional remodeling within hippocampal neuronal populations, particularly in the DG and CA1 regions, relative to aged controls (Fig. 2r,s). GO analysis further identified enrichment of biological processes associated with synaptic plasticity and neuronal development under both conditions, accompanied by coordinated upregulation of associated genes (Fig. 2t,u and Extended Data Fig. 2o). Together, these data indicate that young ovary-derived factors recapitulate, in part, the neurogenic and cognitive benefits observed with young female plasma administration in aged male mice and identify conserved transcriptional signatures across these interventions in the aged male hippocampus.

## Ovary-enriched factor SFRP4 restores cognitive function in the aged male hippocampus

Given that young female plasma more robustly ameliorates age-related cognitive deficits in aged male mice than young male plasma, as well as the pro-youthful cognitive effects of heterochronic young ovary transplantation, we next sought to identify ovary-derived circulating factors associated with female-dependent benefits. To this end, we quantified 11,083 soluble proteins using the SomaScan platform across 39 mouse plasma samples comprising young female, aged female, young male, and aged male groups (Fig. 3a). Comparative proteomic analysis identified 162 proteins with increased abundance in young female plasma relative to young male plasma (Fig. 3b and Extended Data Fig. 3a) and 36 proteins elevated in young female plasma compared to aged female plasma (Fig. 3d and Extended Data Fig. 3b). GO analysis of differentially expressed proteins (DEPs) revealed enrichment of biological processes related to extracellular signaling and tissue remodeling in young female plasma across both comparisons (Fig. 3c,e and Extended Data Fig. 3c,d). Intersection analysis identified 16 proteins—Anti-Müllerian hormone (AMH), Secreted frizzled-related protein 4 (SFRP4), Collagen type II alpha 1 chain (COL2A1), Ephrin type-A receptor 4 (EPHA4), Betacellulin (BTC), Periostin (POSTN), Collagen type V alpha 1 chain (COL5A1), Thrombospondin 3 (THBS3), Urocortin 3 (UCN3), Immunoglobulin superfamily containing leucine-rich repeat protein (ISLR), Collagen type VIII alpha 2 chain (COL8A2), Kelch-like protein 36 (KLHL36), Thrombospondin 4 (THBS4), Secretogranin III (SCG3), Ephrin type-B receptor 6 (EPHB6), and Microfibril-associated glycoprotein 4 (MFAP4)—that were consistently elevated in young female plasma relative to both young male and aged female groups (Fig. 3f). A heatmap of the top-ranked candidates further highlighted a subset of proteins selectively enriched in young female plasma (Fig. 3g).

Analysis of publicly available ENCODE RNA-seq datasets examining tissue-specific expression in C57BL/6 mice revealed that of the factors elevated in young female plasma (Fig. 3g,h and Extended Data Fig. 3e), AMH and SFRP4 were predominantly expressed in the ovary (Fig. 3i,j and Extended Data Fig. 3f). Of note, recent analyses from the UK Biobank linking plasma proteomics to MRI-based imaging-derived phenotypes of brain aging using the ProSig machine-learning framework in humans identified an association between SFRP4 and reduced brain aging and dementia risk^32^. Correspondingly, to initiate functional investigations of individual young ovary-enriched blood factors we elected to focus our studies on SFRP4.

Elevated concentrations of SFRP4 were confirmed in plasma derived from young female mice as well as in aged male mice following heterochronic young ovary transplantation compared to control conditions by Western blot analysis (Fig. 3k–n). Additionally, across young ovary-transplanted and sham aged male mice, higher circulating SFRP4 levels in plasma correlated with improved cognitive performance in both the Y-maze and RAWM tasks (Fig. 3o,p).

To evaluate whether the ovary-enriched factor SFRP4 is sufficient to induce cognitive benefits in the aged male hippocampus, recombinant SFRP4 was administered intravenously to aged male mice, and hippocampal-dependent cognitive function was assessed using the Y-maze, NOR, and RAWM behavioral paradigms following a timeframe consistent with prior plasma administration experiments (Fig. 3q). SFRP4-treated aged male mice showed a preference for the novel arm, but not the novel object, during Y maze and NOR testing (Fig. 3r,s), concordant with observations from heterochronic young ovary transplantation experiments (Fig. 2b,c). During the RAWM training phase, learning progressed similarly between groups, although separation emerged at the final training block (Fig. 3t). This effect coincided with improved performance in the same-day working memory assessment (Fig. 3t). During the RAWM testing phase, SFRP4-treated aged male mice exhibited enhanced spatial memory for platform location compared with control animals (Fig. 3t). General health metrics were comparable between groups (Extended Data Fig. 4a–e). Together, these proteomics and behavioral data identify SFRP4 as an ovary-enriched circulating factor sufficient to promote cognitive benefits in aged male mice.

## Bone-derived osteocalcin mediates cognitive benefits of SFRP4 in aged male mice

To delineate whether the cognitive benefits of SFRP4 arise from either direct central action within the brain or peripheral signaling, we assessed the ability of SFRP4 to cross the blood-brain-barrier (BBB). A subset of female mice received systemic administration of fluorescently labeled SFRP4 (SFRP4–647) (Fig. 4a,b). Whereas SFRP4 was readily detected in plasma, it was absent from the hippocampus, indicating limited brain penetration (Fig. 4c,d). Consequently, we next explored whether its cognitive effects might be mediated indirectly through downstream peripheral signals. To begin, we examined potential links between SFRP4 and previously identified pro-youthful blood factors^9^. Given the established peripheral functions of SFRP4^33–39^, including roles in bone remodeling^40–42^, we selected OCN, a bone-derived hormone, as a candidate downstream mediator due to its ability to cross the BBB^43,44^ and previous implication in regulating hippocampal aging^5,45^. We measured OCN in plasma of aged male mice following systemic SFRP4 administration and observed significantly increased levels by Western blot analysis (Fig. 4f,g). Increased concentrations of OCN were also detected in plasma of aged male mice after heterochronic young ovary transplantation (Fig. 4j,k). Across both SRFP4 treated and young ovary transplantation cohorts, higher circulating OCN levels in aged male mice correlated with improved cognitive performance in the Y-maze and RAWM tasks (Fig. 4h,i,l,m).

Next, we investigated the role of OCN in mediating the cognitive benefits of systemic SFRP4. Aged male mice were systemically administered recombinant SFRP4 or saline together with either an OCN-neutralizing antibody or IgG control antibody intravenously, following the same experimental timeline as our prior SFRP4 administration studies (Fig. 4n). Hippocampal-dependent cognitive function was subsequently assessed using Y-maze and RAWM paradigms (Fig. 4i). Consistent with our previous results, aged male mice administered SFRP4 exhibited a preference for the novel arm during Y maze testing and decreases errors in locating a hidden platform during RAWM testing compared to saline treated aged mice (Fig. 4o,p). However, neutralization of OCN abolished the SFRP4-induced improvements in working memory and spatial memory compared to aged male mice treated with SFRP4 and receiving IgG control (Fig. 4o,p). No difference in health metrics was observed between groups (Extended Data Fig. 5a–c). These behavioral data identify OCN as a downstream peripheral mediator of the cognitive benefits of increased systemic SFRP4 in aged male mice.

## Discussion

Collectively, our data show that female-associated cognitive resilience can be transferred to the aged male brain through systemic factors derived from the young ovary. We identified ovary-enriched SFRP4 as one such blood-borne factor that promotes cognitive benefits in aged males through induction of the bone-derived rejuvenating hormone OCN. These results define an ovary-bone-brain axis through which blood factors confer female cognitive resilience in aging.

Emerging studies point to extensive communication between peripheral organs—including muscle, liver and bone—and the brain in regulating cognition during aging^5–7^. Our findings build upon this framework by highlighting the sex-transcending benefits of young ovary-derived factors in rescuing cognitive impairments in the aged male brain. Moreover, the observation that female cognitive advantages are most pronounced in women prior to menopause^46^ underscores the idea that ovarian-derived signals contribute to protection against age-associated cognitive decline. Notably, prior studies have shown that while young ovariectomized female mice exhibit cognitive deficits associated with sex-hormone loss^47,48^, administration of sex hormones alone in males fails to recapitulate cognitive benefits^26^, indicating that ovary-derived factors beyond classical hormones also contribute to sex differences in cognitive resilience. In this context, identification of SFRP4 as a pro-youthful factor and its association with increased circulating OCN, posit an ovary-bone-brain signaling axis that links reproductive biology, skeletal endocrine signaling, and cognition. Together, these findings suggest that leveraging ovary-derived systemic resilience pathways present in young females may represent a strategy to mitigate cognitive decline across sexes during aging.

We note that while systemic administration of young female blood plasma reversed age-associated cognitive deficits across all hippocampal-dependent behavioral paradigms, heterochronic young ovary transplantation and systemic SFRP4 administration recapitulated select cognitive benefits. This divergence suggests that the rejuvenating influence of young female plasma extends beyond ovary-derived circulating signals and likely reflects contributions from additional peripheral sources. Consistent with this interpretation, eight of the ten circulating factors enriched in young female plasma show predominant expression in tissues outside the ovary, supporting the existence of non-ovarian systemic contributors. Concordantly, thrombospondin-4 (THBS4), enriched in young female plasma compared with young male and aged female plasma, has been identified as a pro-youthful factor that enhances synaptic plasticity in human embryonic stem cell-derived neurons *in vitro*^49^. Collectively, these findings support a model in which female-associated cognitive resilience arises from coordinated systemic cues originating across multiple tissues and conveyed through blood.

From a translational perspective, recent machine learning-based analysis from the UK biobank has identified neuroprotective associations between SFRP4 plasma levels and reduced dementia risk in humans^32^. However, despite these promising observations, no functional studies investigating the effects of systemic SFRP4 on cognition in aging or neurodegenerative disease contexts had to date been conducted. In this respect, our data raise the exciting possibility that the beneficial effects of increasing systemic SFRP4 late in life may be broad reaching, extending to age-related cognitive decline and neurodegenerative disease conditions across sexes in the elderly.

## Methods

### Animal Models.

The following mouse strains were used: C57BL/6 aged mice (The Jackson Laboratory) and C57BL/6 aged mice (National Institute on Aging). Sample sizes were determined using standard power calculations with α = 0.05 and a power of 0.8. Power and sample sizes were estimated using an online tool (http://www.stat.uiowa.edu/~rlenth/Power/index.html), based on prior experience with the respective behavioral assays, assay variability, and inter-individual differences within groups. Within each experiment, animals from the same strain and vendor that were aged together were used. Mice from Jackson were acquired at 2 months of age and aged in-house throughout their lifespan. For behavioral testing, animals were transferred to designated behavioral testing facilities. Behavioral assessments were conducted at two locations on the UCSF Parnassus campus: the UCSF Rehabilitation Behavioral Core and the Villeda Lab Behavioral Suite. Mice were housed under specific pathogen–free conditions on a 12-h light–dark cycle. All animal handling and experimental procedures were performed in accordance with institutional guidelines approved by the University of California, San Francisco Institutional Animal Care and Use Committee (IACUC).

### Animal Plasma Collection and Administration.

Mouse plasma was collected by intracardiac bleeding at the time of sacrifice from 60 young male and 60 young female mice. Blood was collected in EDTA-coated tubes, and plasma was isolated by centrifugation at 1,000 × g. Plasma samples from individual mice were maintained separately for western blot and SomaScan analyses or pooled for plasma administration experiments. Plasma was aliquoted and stored at −80 °C until use. Prior to administration, plasma was dialyzed against PBS using 3.5-kDa D-Tube dialyzers (EMD Millipore) to remove EDTA. Aged mice were systemically treated with plasma (100 μl per injection) isolated from young male or female mice via intravenous tail vein injection eight times over 24 days.

### Ovary Transplantation.

Ovary transplantation surgery was performed as previously described^31,50^. Ovaries were collected from 2-month-old female donor mice under isoflurane anesthesia (2–3% via inhalation). Following anesthesia, a dorsal skin incision was made and bilateral ovariectomies were performed to harvest the ovaries. Excised ovaries were immediately placed in chilled DMEM (Thermo Fisher Scientific, Cat# 10569044) and kept on ice until transplantation. Recipient mice consisted of aged male mice (22 months old) anesthetized with isoflurane (2–3%). Two donor ovaries were implanted subcutaneously into each recipient mouse, with donor and recipient animals matched at a 1:1 ratio. Grafts were secured to the subcutaneous tissue using 8–0 nonabsorbable nylon sutures (Ethicon, Cat# 2815G) to promote graft stability and recovery. Surgical incisions were closed using wound clips (Fisher Scientific, Cat# 018045). Postoperative care included subcutaneous administration of carprofen, enrofloxacin, and buprenorphine for analgesia and infection prophylaxis according to recommended dosing guidelines. Animals were monitored closely during the postoperative recovery period. General health and recovery were evaluated at multiple time points after surgery by measuring body weight and assessing maintenance behaviors, including grooming activity.

### Novel Object Recognition (NOR).

Recognition memory was evaluated using the novel object recognition (NOR) paradigm as previously described^6^. During the habituation phase (day 1), mice were placed in an empty arena and allowed to freely explore for 10 min. Locomotor activity was recorded through infrared photobeam interruptions and analyzed using MotorMonitor software (Kinder Scientific). On day 2 (training phase), two identical objects were positioned in the arena, and mice were permitted to explore them for 5 min. On day 3 (testing phase), one of the familiar objects was replaced with a novel object, and mice were again allowed to explore the arena for 5 min Exploration time for each object was quantified using EthoVision XT tracking software (Noldus Information Technology). Two distinct object sets were used across experiments. To minimize potential bias arising from inherent object preference, half of the animals encountered object A as the novel object, whereas the remaining animals encountered object B. To further control for positional bias, the location of the novel object within the arena was counterbalanced across mice. Recognition performance was expressed as a preference index calculated as the time spent interacting with the novel object divided by the total time spent interacting with both objects, multiplied by 100. Animals that failed to interact with both objects during the training phase were excluded from subsequent analyses.

### Y-maze.

Spatial working memory was evaluated using a Y-maze forced alternation paradigm. During the training session, mice were introduced into the start arm facing the wall and allowed to freely explore the start arm and one designated familiar arm for 5 min, while access to the third arm (novel arm) was blocked. To eliminate potential olfactory cues, the maze was thoroughly cleaned between trials, and the identity of the familiar arm was alternated across mice. After the training phase, animals were returned to their home cages. Following a 35–45 min interval, mice were reintroduced into the start arm and allowed to explore all three arms of the maze for 5 min. The duration spent in each arm was quantified using EthoVision XT tracking software (Noldus Information Technology). Arm occupancy was expressed as the percentage of time spent in each arm relative to the total time spent in all arms during the first minute of the test session. A discrimination index was calculated as (entries into the novel arm − entries into the familiar arm) / (entries into the novel arm + entries into the familiar arm).

### Radial Arm Water Maze.

Spatial learning and memory were assessed using the radial arm water maze (RAWM) paradigm according to established protocols^51^. In this task, mice are trained to locate a constant goal arm throughout the training and testing phases, while the start arm changes with each trial. Entry into an incorrect arm is scored as an error, and errors are averaged across training blocks (three consecutive trials per block). During training (day 1), mice undergo 12 trials (blocks 1–4), with trials alternating between a visible and a hidden platform. Following a 1-h break, learning is assessed in three trials (block 5) using only a hidden platform. During testing (day 2), mice are tested in 15 trials (blocks 6–10) with a hidden platform. Investigators were blinded to treatment groups during scoring.

### Health Metrics.

Nest-building behavior was evaluated using the nestlet assay as previously described^52^. Mice were supplied with two compressed cotton nestlets and allowed up to 48 h to construct a nest. Nest-building performance was quantified using a five-point scoring scale, where a score of 1 indicated no nest construction and a score of 5 indicated a fully enclosed nest, according to established criteria. To improve measurement reliability, nesting behavior for each mouse was assessed in two independent trials conducted one week apart, and the resulting scores were averaged for analysis.

### Tissue Collection.

Mice were deeply anesthetized with ketamine (87.5 mg kg^−1^) and xylazine (12.5 mg kg^−1^) and transcardially perfused with 25 ml of ice-cold phosphate-buffered saline (PBS). For animals used in histological analysis of the brain, PBS perfusion was followed by perfusion with 25 ml of ice-cold 4% paraformaldehyde (PFA). In a subset of PBS-perfused mice, peripheral tissues including liver, heart, kidney, spleen, lung, and tibialis anterior muscle were rapidly dissected and snap-frozen. Brains were bisected along the sagittal plane. From one hemisphere, the hippocampus were microdissected and immediately snap-frozen for molecular analyses. The contralateral hemisphere was post-fixed in 4% paraformaldehyde in phosphate buffer (pH 7.4) at 4 °C for 48 h, followed by cryoprotection in 30% sucrose.

### Immunohistochemistry.

Tissue processing and immunostaining were performed on free-floating sections using standard published techniques^53^. Cryoprotected brains were sectioned coronally at 40 μm using a freeze-stage microtome (Leica). Sections were first permeabilized in pre-treatment buffer containing 0.2% Triton X-100 in TBST for 30 min, followed by three washes in TBST and blocking in TBST supplemented with 3% normal donkey serum (NDS). Sections were then incubated overnight at 4 °C with primary antibody against DCX (Cell Signal Technology Cat# 40619S) diluted in TBST containing 3% NDS. Following primary antibody incubation, sections were washed and incubated with appropriate secondary antibodies (1:500 dilution in TBST + 3% NDS) for 1 h at room temperature. Nuclei were counterstained with Hoechst (Thermo Fisher Scientific, Cat# H3570). Sections were mounted onto Superfrost Plus microscope slides and coverslipped using ProLong Gold mounting medium. Images were acquired using confocal microscopes (Zeiss LSM800 or Zeiss LSM900) or a bright-field microscope (Keyence). Labeling intensity and thresholded areas were quantified using FIJI and Zeiss Zen image analysis software and averaged across 3–5 hippocampal sections per mouse.

### Single-nucleus RNA sequencing.

Single-nucleus RNA sequencing (snRNA-seq) was performed on hippocampal nuclei from four independent mice per experimental group, with each mouse processed as a separate biological replicate through nuclei isolation, FANS, library preparation, and sequencing using a modified version of the protocol recommended by 10x Genomics. Only male mice were included in the snRNA-seq analyses. Briefly, flash-frozen hippocampi were homogenized in 500 μL Nuclei EZ Prep lysis buffer (Sigma-Aldrich, NUC101) supplemented with RNase inhibitor using a glass Dounce homogenizer (Wheaton, Cat# 357538) with 20 strokes of the loose pestle followed by 20 strokes of the tight pestle. An additional 500 μL of lysis buffer was then added and samples were incubated on ice for 7 min. Homogenates were filtered through a 40-μm cell strainer and centrifuged at 500 × g for 5 min at 4 °C. Pelleted nuclei were resuspended in 1 mL wash buffer (PBS containing 1% BSA and RNase inhibitor) and incubated on ice for 5 min. After a second centrifugation step (500 × g, 5 min, 4 °C), the supernatant was removed and nuclei were resuspended in 400 μL wash buffer containing Hoechst 33342 (1:10,000 dilution). Following a 5-min incubation, nuclei were filtered through a 35-μm FACS tube filter and subjected to fluorescence-activated nuclei sorting (FANS). Nuclei were sorted on a BD FACSAria II using a 100-μm nozzle at a flow rate of 1–2.5. Sorting gates were first established using forward and side scatter parameters, followed by exclusion of doublets based on height and width parameters. Hoechst-positive nuclei from each mouse were collected separately and were not pooled across animals. Purified nuclei were submitted to the UCSF CoLab Genomics Core for library preparation using the Chromium Single Cell 3′ Gene Expression platform (10x Genomics). Approximately 30,000 nuclei were loaded per biological replicate for single-nucleus capture. Separate cDNA libraries were generated for each mouse according to the manufacturer’s protocol. Final libraries were sequenced on either the NovaSeq 6000 or NovaSeq X (10B flow cell) platforms at the UCSF CAT Core.

### Single nucleus RNA sequencing analysis.

Raw sequencing reads were aligned to the mouse reference genome (GRCm39–2024-A) using CellRanger (v9.0.1 To reduce technical noise arising from ambient RNA contamination in droplet-based platforms, raw count matrices were corrected using CellBender (v0.3.0)^54^. Potential doublets were subsequently identified and removed with Scrublet algorithm^55^. Quality control filtering was then applied to exclude low-quality nuclei. Nuclei were retained if they contained between 1,000 and 50,000 total UMI counts and between 500 and 10,000 detected genes. To account for RNA degradation and mitochondrial bias, nuclei with ribosomal RNA content greater than 40% or mitochondrial RNA content greater than 7% were excluded. After filtering, a total of 109,922 nuclei (saline), 54,773 nuclei (young male plasma), 91,995 nuclei (young female plasma), 70,514 nuclei (sham), and 98,585 nuclei (ovary transplantation) were retained for downstream gene expression analysis. Downstream preprocessing and dataset integration were performed using Scanpy (v1.10.2) and scVI-tools (v1.1.5)^56^, with model parameters optimized for each dataset. scVI was used to learn a latent representation for batch correction and downstream analyses. Cell clusters were identified using the Leiden algorithm. Cell types were annotated using CellTypist (v1.6.3)^57^ and further validated by examining the expression of established cell type–specific marker genes. Differential gene expression analysis was performed using Scanpy’s rank_genes_groups function with the Wilcoxon rank-sum test. Genes were ranked by log fold change and statistical significance. Gene Ontology (GO) enrichment analysis was performed using GSEApy (v1.1.3) through the Enrichr API with the GO Biological Process and Cellular Component gene set libraries.

### SomaScan assay.

Plasma proteomic profiling was performed using the SomaScan 11K assay (SOW 2.5; SomaLogic/Standard BioTools), an aptamer-based platform that uses slow off-rate modified DNA aptamers (SOMAmer reagents) to quantify protein abundance in plasma samples^58^. EDTA-plasma samples from young male (n = 10), young female (n = 10), aged male (n = 10), and aged female (n = 9) mice were analyzed. Assays were performed by SomaLogic/Standard BioTools using the standard vendor normalization and quality-control pipeline. Protein abundance values were reported as relative fluorescence units (RFUs) and used for downstream analyses.

### Western Blot Analysis.

Plasma samples were mixed with 4× NuPAGE LDS sample buffer containing water and β-mercaptoethanol. One microliter of plasma was loaded per lane, separated on 4–12% SDS–polyacrylamide gradient gels (Bio-Rad, Cat# 11346–02), and transferred onto nitrocellulose membranes using the Trans-Blot Turbo transfer system (Bio-Rad). Following transfer, membranes were occasionally cut into strips at approximately 25 kDa and 75 kDa to enable parallel probing of proteins with different molecular weights. Membranes were blocked in 5% milk in Tris-buffered saline containing 0.1% Tween-20 (TBST) and incubated with primary antibodies against SFRP4 (Abcam, Cat# ab154167) or osteocalcin (Cell Signaling Technology, Cat# 59757S). Protein signals were detected using horseradish peroxidase–conjugated secondary antibodies and enhanced chemiluminescence (ECL) substrate (Bio-Rad, Cat# 1705060). Membranes were imaged using a ChemiDoc imaging system (Bio-Rad), and band intensities were quantified using the gel analysis tools in FIJI/ImageJ, with Ponceau staining used to verify comparable protein loading across lanes.

### Exogenous SFRP4 Administration and Osteocalcin Neutralization.

For SFRP4 treatment experiments, recombinant mouse SFRP4 (0.5 μg; R&D Systems, Cat# 9806-SF-050) or saline was administered via intravenous injection (100 μl per injection) eight times over a 24-day period prior to behavioral testing. Given that circulating SFRP4 levels in young female plasma were comparable to those previously reported for the pro-youthful factor PF4 in young plasma, we adopted a similar systemic dosing paradigm^8^ (0.5 μg per injection, eight injections over 24 days). To inhibit osteocalcin (OCN) activity, mice received intravenous injections of an anti-OCN antibody (200 ng; Santa Cruz Biotechnology, Cat# sc-365797) or control mouse IgG (200 ng; Santa Cruz Biotechnology, Cat# sc-2025) following the same dosing schedule (eight injections over 24 days) before behavioral assessments. The anti-OCN antibody dose was selected based on a previously established *in vivo* OCN-neutralization protocol^59^

### Labelled SFRP4 and Transferrin Blood–Brain Barrier Permeability Assay.

Recombinant SFRP4 (50 μg) was dissolved in 100 μl sterile PBS. Holo-transferrin (Sigma, Cat# T4132) was prepared at 50 mg in 4 ml sterile PBS. Atto 647N NHS ester (Sigma, Cat# 18373–1MG-F) was dissolved in DMSO to generate a 30 mM stock solution. Recombinant SFRP4 or transferrin was conjugated with Atto 647N NHS ester at a molar ratio of 1:1.8 and incubated for 90 min at room temperature with constant agitation. The labeling reaction was quenched by adding 50 mM Tris buffer (pH 8.0) followed by a 10 min incubation at room temperature. Unbound dye was removed using Zeba spin desalting columns (7 kDa MWCO; Thermo Fisher Scientific, Cat# 89892), with two sequential purification steps performed using two columns per round. Protein concentration of the purified conjugates was determined using a NanoDrop spectrophotometer, adjusted to 10 mg ml^−1^ in PBS, and stored at −80 °C until use. Successful fluorescent labeling was confirmed by western blot analysis. Briefly, 1 μg of labeled and unlabeled SFRP4 or transferrin was separated on 4–12% NuPAGE Bis-Tris gels, transferred onto nitrocellulose membranes, and fluorescence signals were detected using a ChemiDoc imaging system (Bio-Rad). For in vivo blood–brain barrier permeability experiments, 0.1 mg of labeled SFRP4 (SFRP4–488) and 1 mg of labeled transferrin (TF-488) were administered intravenously 20 h prior to tissue collection. Mice were anesthetized with ketamine (87.5 mg kg^−1^) and xylazine (12.5 mg kg^−1^) and transcardially perfused with 25 ml ice-cold PBS followed by 25 ml of 4% paraformaldehyde (PFA). Brains were postfixed in 4% PFA at 4 °C for 48 h and cryoprotected in 30% sucrose. Tissue was sectioned coronally at 40 μm using a freeze-stage microtome (Leica). Sections were stored in cryoprotective medium until imaging. Hippocampal sections were imaged using confocal microscopy (Zeiss LSM900 or LSM800) and analyzed with Zeiss Zen and ImageJ software. Fluorescence intensity of SFRP4–488 and TF-488 was quantified within vascular regions of the hippocampus defined by vascular labeling.

### Data and Statistical Analyses.

All experiments were conducted in a randomized and blinded manner by an independent investigator. Researchers remained blinded during histological, biochemical, and behavioral analyses. Experimental groups were revealed only after completion of data collection and statistical analysis. Data are presented as mean ± s.e.m. Normality of the data distribution was assessed using either the D’Agostino–Pearson omnibus test or the Shapiro–Wilk test. Statistical analyses were performed using Prism versions 8–10 (GraphPad Software). Comparisons between two groups were conducted using two-tailed unpaired Student’s t-tests. For experiments involving more than two groups, differences among group means were evaluated using one-way ANOVA followed by the appropriate post hoc multiple-comparison test, as specified in the figure legends. Additional statistical information for individual experiments is provided in the corresponding figure legends. All data generated or analyzed during this study are included in this published article.

## Extended Data

**Extended Data Fig. 1.**
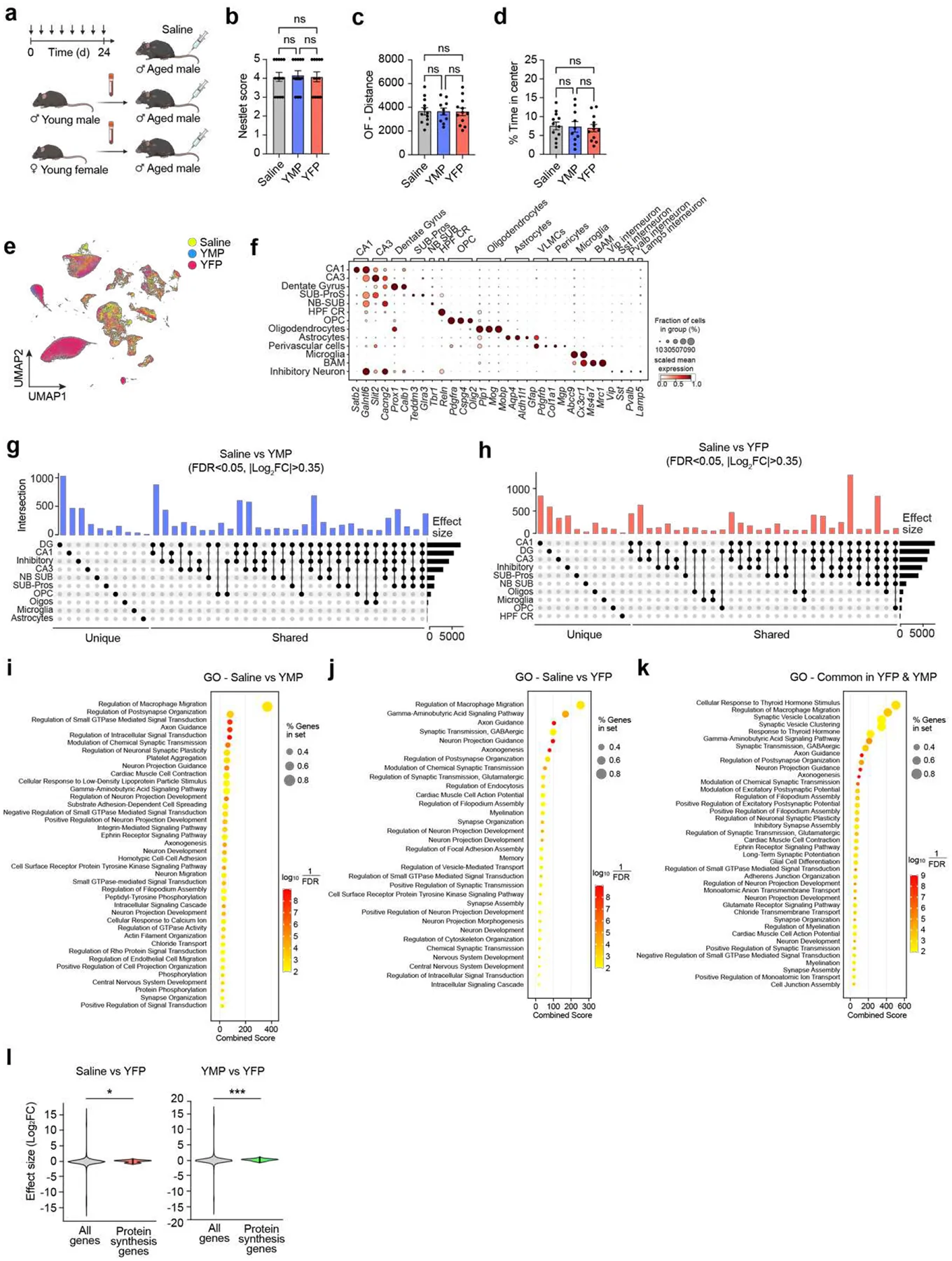
Behavioral health metrics and hippocampal snRNA-seq gene signatures of saline-, young male plasma-, and young female plasma-treated aged male mice. (**a**) Behavioral and health metrics were assessed in aged (22 months) male mice treated with saline, young male plasma (YMP), or young female plasma (YFP). (**b**) Hippocampal-dependent nest-building performance was scored on a scale from 1 (worst) to 5 (best) (n=12–13 mice per group). (**c,d**) Locomotor activity (C, total distance traveled) and anxiety-like behavior (d, % time in center) were measured using the open field (OF) test, in which mice freely explored an empty arena for 10 min (n=10–12 mice per group). (**e**) UMAP of hippocampal nuclei showing cell origin by treatment group. (**f**) Dot plots showing representative marker genes for each cell type identified by snRNA-seq of hippocampi from saline-, YMP-, or YFP-treated aged mice. (**g**) UpSet plot showing shared and unique DEGs by cell type for YMP versus saline (FDR<0.05, |log_2_FC|>0.35). (**h**) UpSet plot showing shared and unique DEGs by cell type for YFP versus saline (FDR<0.05, |log_2_FC|>0.35). (**i**) GO biological processes associated with parenchymal DEGs for YMP versus saline. (**j**) GO biological processes associated with parenchymal DEGs for YFP versus saline. (**k**) GO biological processes associated with conserved parenchymal DEGs. Data are shown as mean ± s.e.m. Statistical analyses were performed using paired t-test with Mann-Whitney test one-sample t-tests (b), one-way ANOVA followed by Šidák’s multiple comparisons test (c,d). (**l**) Effect size of protein synthesis genes in YFP versus saline or YMP hippocampus compared with all genes in the dataset (Differences in effect size distributions were assessed using two-sided Mann–Whitney U tests; asterisk indicates FDR<0.001).

**Extended Data Fig. 2.**
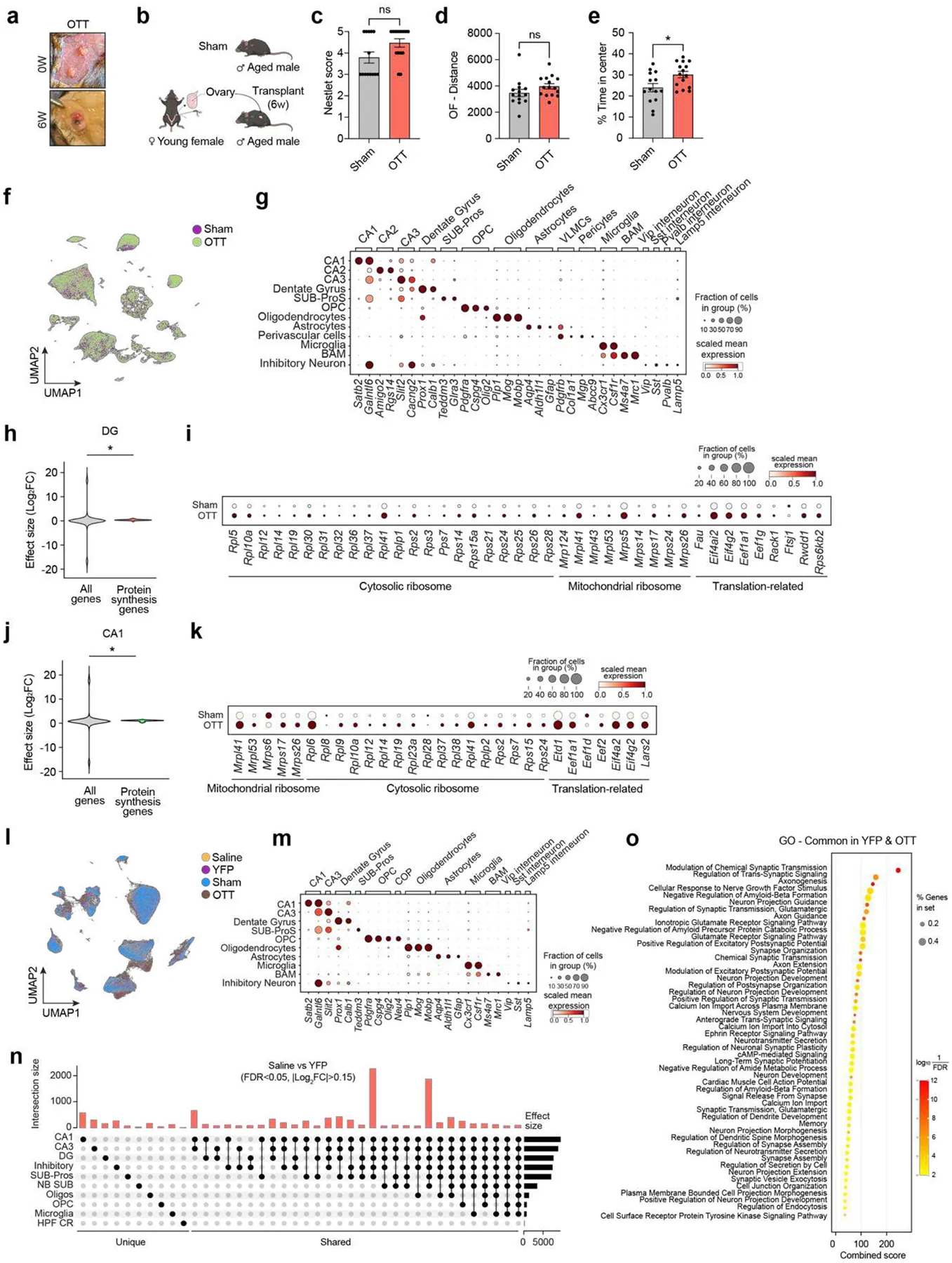
Behavioral health metrics and hippocampal snRNA-seq transcriptional profiles following sham surgery or ovary transplantation in aged male mice. **(a)** Images of ovaries immediately after transplantation and 6 weeks after transplantation. Ovaries remained viable and vascularized 6 weeks after transplantation and behavioral testing. **(b)** Behavioral and health metrics were assessed in sham- or ovary-transplanted (OTT) aged (22 months) male mice. **(c)** Hippocampal-dependent nest-building performance was scored on a scale from 1 (worst) to 5 (best) (n=14–15 mice per group). **(d, e)** Locomotor activity (d, total distance traveled) and anxiety-like behavior (e, % time in center) were measured using the open field (OF) test, in which mice freely explored an empty arena for 10 min (n=10–12 mice per group). **(f)** UMAP of hippocampal nuclei showing cell origin by treatment group (sham or OTT). **(g)** Dot plots showing representative marker genes for each cell type identified by snRNA-seq of hippocampi from sham- or OTT-treated aged mice. **(h,j)** Effect size of protein synthesis genes in the DG (h) and CA1 (j) of OTT versus sham hippocampus compared with all genes in the dataset (asterisk indicates FDR<0.001). **(I,k)** Dot plots of ribosomal DEGs from protein synthesis–related GO terms in the DG (i) and CA1 (k). **(l)** UMAP of hippocampal nuclei showing cell origin by treatment group (saline, YFP, sham, or OTT). **(m)** Dot plots showing representative marker genes for each cell type identified by snRNA-seq of hippocampi from saline-, YFP-, sham-, or OTT-treated aged mice. **(n)** UpSet plot showing shared and unique DEGs by cell type for YFP versus saline (FDR<0.05, |log_2_FC|>0.15). **(o)** GO biological processes associated with conserved parenchymal DEGs. Data are shown as mean ± s.e.m. Statistical analyses were performed using paired t-test with Mann-Whitney test one-sample t-tests (c), unpaired t-tests with Welch’s correction (d,e). *P < 0.05

**Extended Data Fig. 3.**
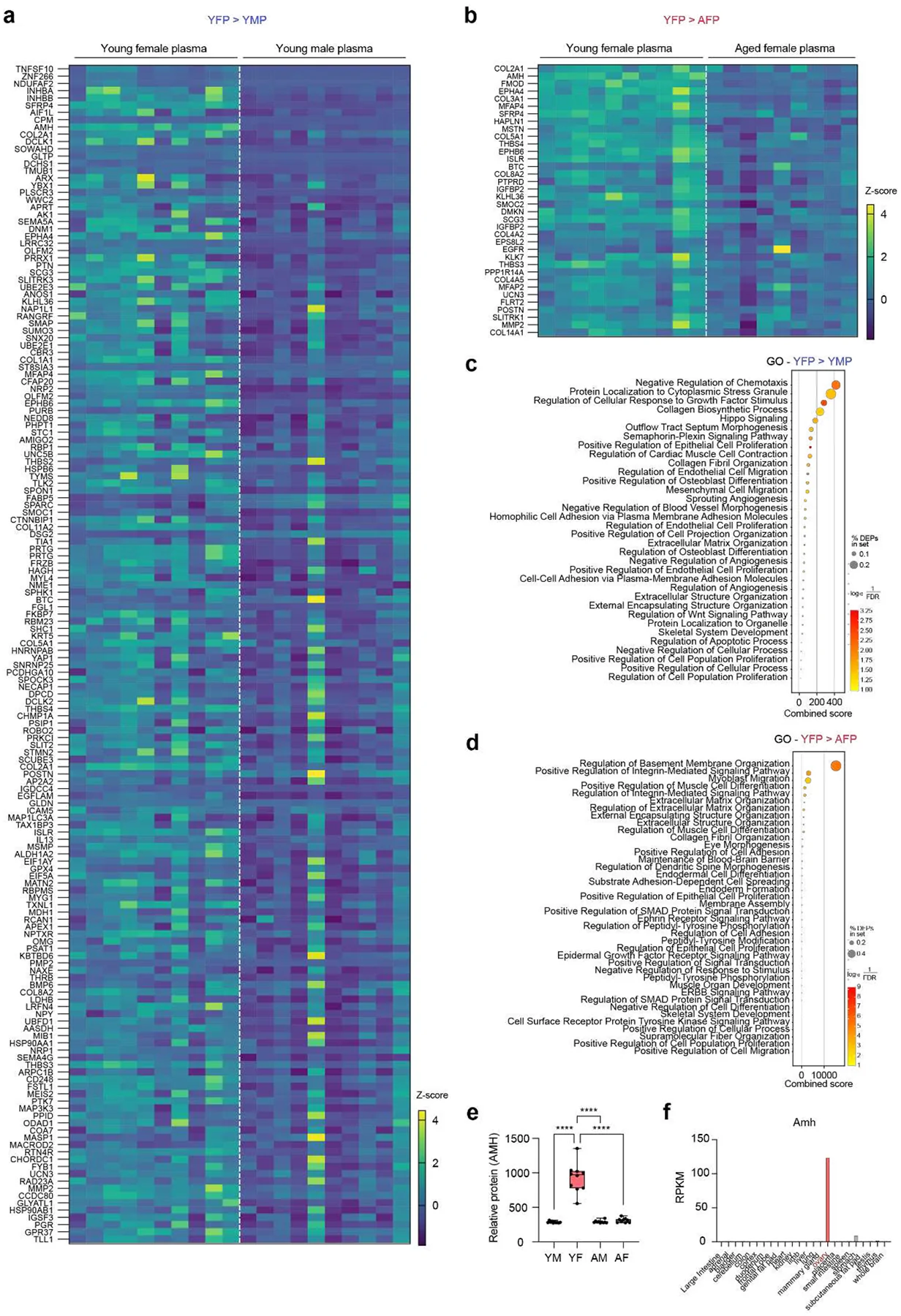
Young female plasma exhibits increased levels of proteins compared to male and aged female plasma. **(a)** Matrix plots of the 162 differentially expressed proteins (DEPs) in plasma derived from young female compared to male mice. **(b)** Matrix plots of the 36 DEPs in plasma derived from young compared to aged female mice. **(c)** Gene Ontology (GO) biological processes associated with plasma DEPs for young female plasma (YFP) versus young male plasma (YMP). **(d)** GO biological processes associated with plasma DEPs for young female plasma (YFP) versus aged female plasma (AFP). **(e)** Bar graphs showing AMH levels from SomaScan assay results. **(f)** Bar graphs showing the expression of *Amh* across mouse tissues based on ENCODE transcriptomic datasets (C57BL/6 mice). Data are shown as mean ± s.e.m. unless otherwise indicated. Panel e is presented as a box-and-whisker plot (median, interquartile range, and min–max). Statistical analyses were performed using one-way ANOVA followed by Tukey’s multiple comparisons test (e). ****P < 0.0001

**Extended Data Fig. 4.**
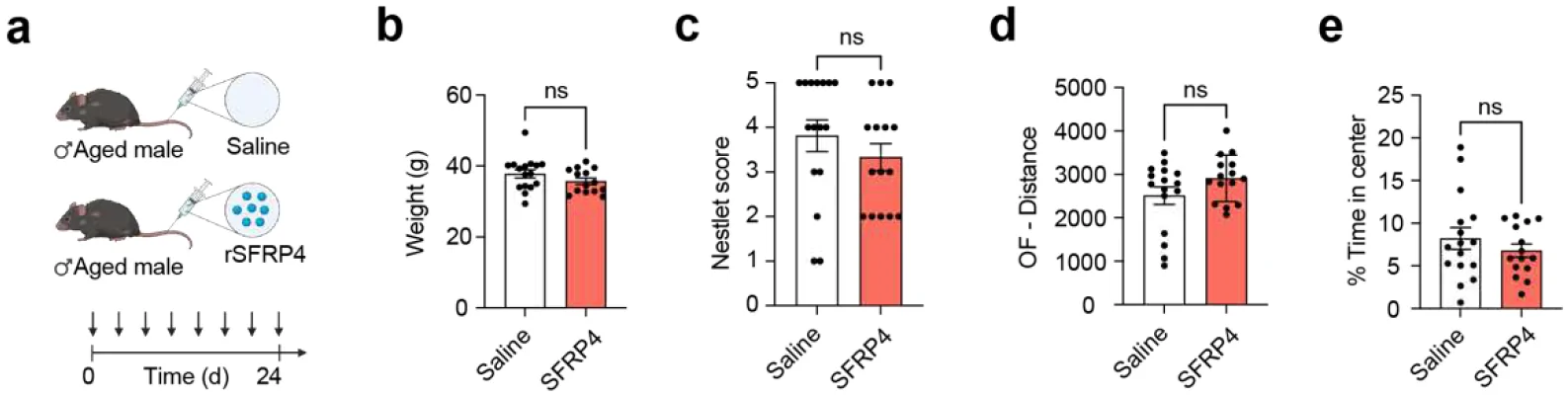
Systemic administration of ovary-enriched factor SFRP4 does not alter general health metrics in aged male mice. **(a)** Behavioral and health metrics were assessed in saline- or recombinant SFRP4- treated aged (22 months) male mice. **(b)** Body weight measurements. **(c)** Hippocampal-dependent nest-building performance was scored on a scale from 1 (worst) to 5 (best) (n=15–16 mice per group). **(d, e)** Locomotor activity (I, total distance traveled) and anxiety-like behavior (J, % time in center) were measured using the open field (OF) test, in which mice freely explored an empty arena for 10 min (n=15–16 mice per group). Data are shown as mean ± s.e.m. unless otherwise indicated. Statistical analyses were performed using paired t-test with Mann-Whitney test (c) and unpaired t-tests with Welch’s correction (b,d,e).

**Extended Data Fig. 5.**
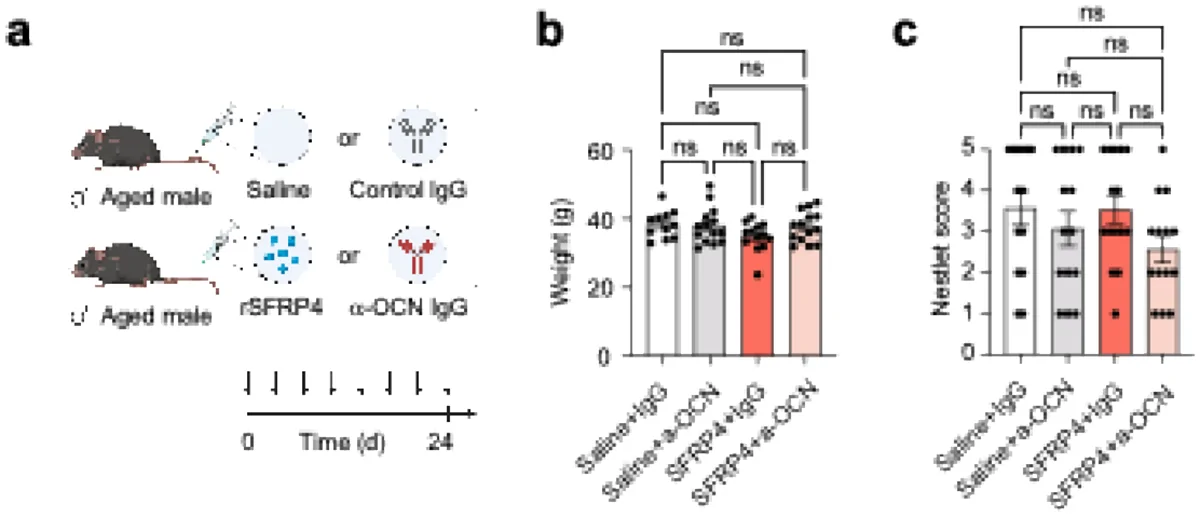
Osteocalcin does not alter general health metrics in aged male mice. **(a)** Behavioral and health metrics were assessed in aged (22 months) male mice following administration of recombinant SFRP4 (5 μg/ml) and an anti-OCN antibody (200 ng). **(b)** Body weight measurements. **(c)** Hippocampal-dependent nest-building performance was scored on a scale from 1 (worst) to 5 (best) (n=11–14 mice per group). Data are shown as mean ± s.e.m. Statistical analyses were performed using one-way ANOVA followed by Tukey’s multiple comparisons test (b,c)

## Supplementary Material

**Supplementary Data 1. Processed SomaScan proteomic data.** Normalized protein expression values (RFU) for each sample are provided. Rows correspond to individual samples annotated by age and sex (young female, aged female, young male, aged male), and columns correspond to protein targets with associated annotation (UniProt ID, gene symbol, and protein name).

Supplementary Files

This is a list of supplementary files associated with this preprint. Click to download.

- HuhNatureSupplementaryData1.xlsx

## Figures and Tables

**Figure 1.**
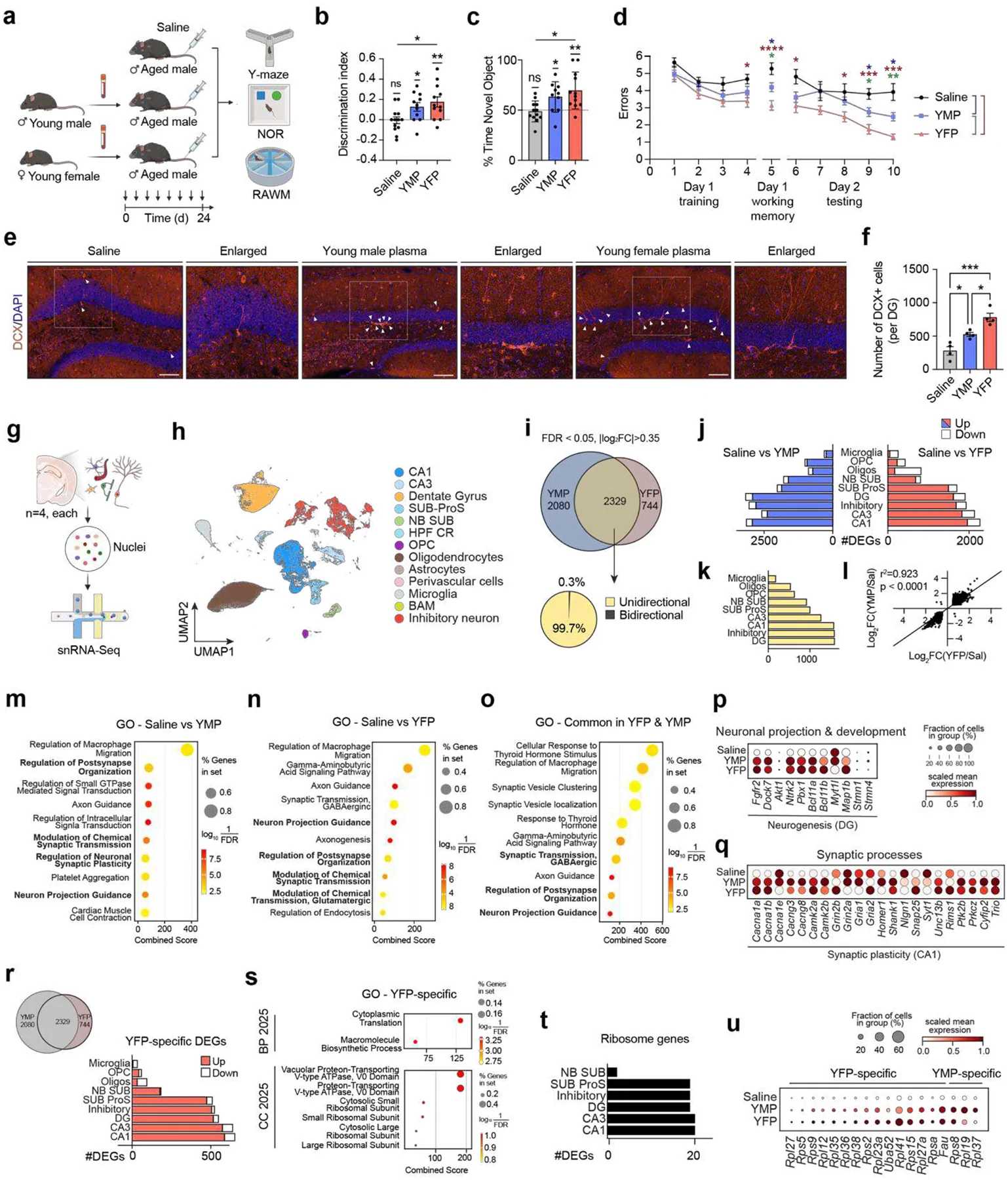
Systemic administration of young female plasma induces greater hippocampal enhancements that young male plasma in aged male mice. **(a)** Plasma from young (3-month-old) male or female mice was administered intravenously to aged (22-month-old) male mice eight times over 24 days (100 μl per injection), followed by cognitive testing. **(b–d)** Hippocampal-dependent cognitive function assessed by Y-maze discrimination index (b), novel object recognition (NOR) preference index (c), and radial arm water maze (RAWM) errors (d) following saline, young male plasma (YMP), or young female plasma (YFP) treatment (n = 11–13, 10–12, and 12 mice per group, respectively). **(e,f)** Representative images and quantification of Doublecortin (DCX) immunolabelling in the dentate gyrus (DG) (n = 4 mice per group; arrowheads, individual cells; scale bar, 200 μm). **(g)** Hippocampal single-nucleus RNA sequencing (snRNA-seq) following saline, YMP or YFP treatment (n = 4 mice per group). **(h)** UMAP showing the identified hippocampal cell types in the snRNA-seq dataset. **(i)** Overlap of parenchymal differentially expressed genes (DEGs) in YMP and YFP versus saline, including conserved unidirectional DEGs. **(j–l)** Cell-type distribution and correlation of conserved parenchymal DEGs. **(m–o)** Top Gene Ontology (GO) biological processes associated with YMP, YFP and conserved DEGs. **(p,q)** Neurogenesis-related DG and synaptic plasticity-related CA1 DEGs. **(r,s)** Identification and GO analysis of YFP-specific parenchymal DEGs. **(t,u)** Cell-type distribution of ribosomal DEGs and protein synthesis-related genes. Data are shown as mean ± s.e.m. Statistical analyses were performed using one-sample t-tests versus 0 (b) or 50% (c) (within-group comparisons) and one-way ANOVA followed by Tukey’s multiple comparisons test (b–d, between-group comparisons), and one-way ANOVA followed by Šidák’s multiple comparisons test (f). *P < 0.05, **P < 0.01, ***P < 0.001, ****P < 0.0001.

**Figure 2.**
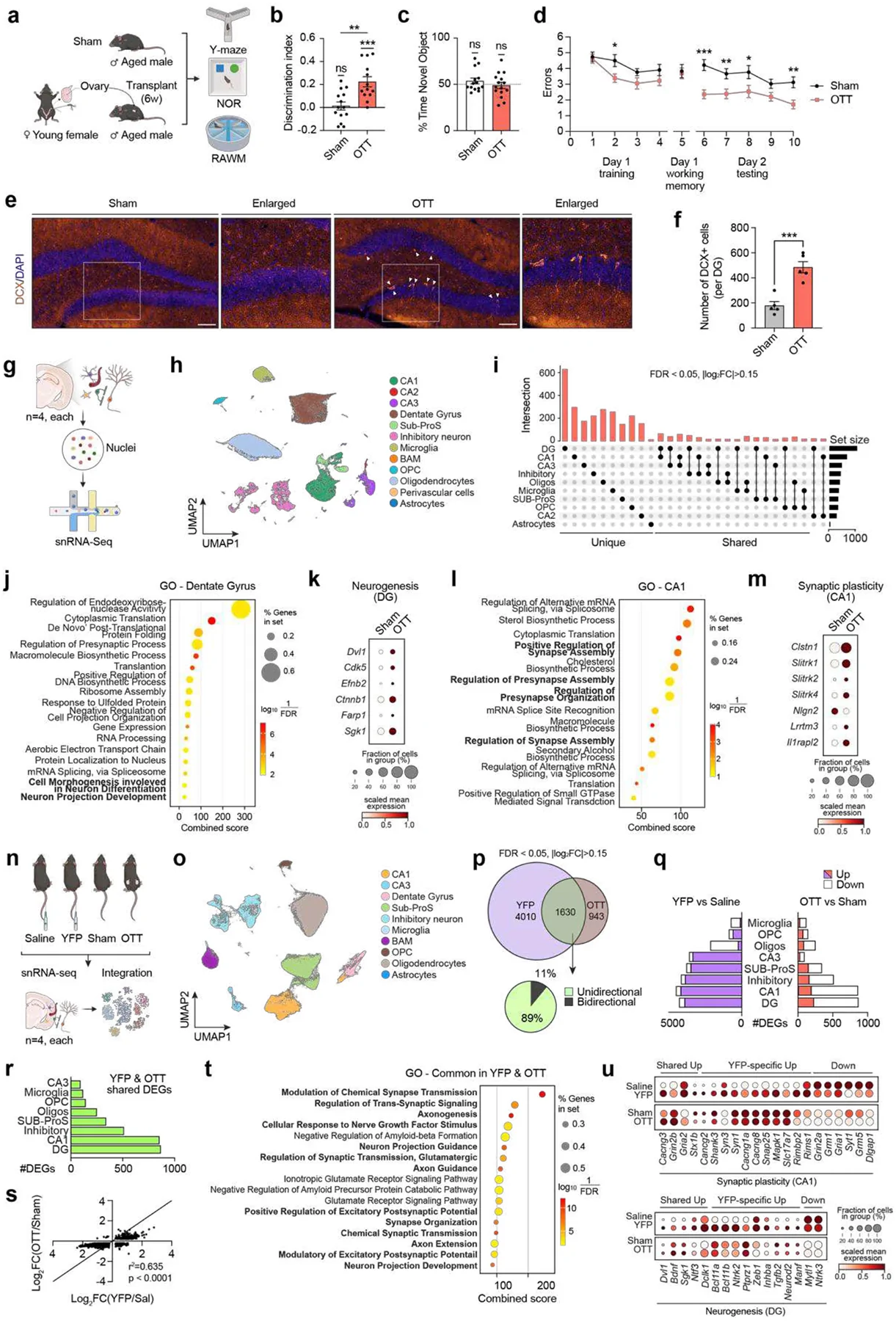
Heterochronic young ovary transplantation induces hippocampal improvements in aged male mice. **(a)** Experimental design showing heterochronic transplantation of ovaries from young (3-month-old) female mice into aged (22-month-old) male mice, followed by cognitive testing. **(b)** Spatial working memory assessed by Y-maze discrimination index (n = 13–14 mice per group). **(c)** Object recognition memory assessed by novel object recognition (NOR) preference index (n = 14–15 mice per group). **(d)** Hippocampal-dependent spatial learning and memory assessed by radial arm water maze (RAWM) errors (n = 13–14 mice per group). **(e,f)** Representative images and quantification of Doublecortin (DCX) labelling in the dentate gyrus (DG) (n = 5 mice per group; arrowheads, individual cells; scale bar, 200 μm). **(g)** Hippocampal single-nucleus RNA sequencing (snRNA-seq) following sham surgery or ovary transplantation (OTT) (n = 4 mice per group). **(h)** UMAP of identified hippocampal cell types. **(i)** UpSet plot showing shared and unique parenchymal differentially expressed genes (DEGs) across cell types. **(j–m)** Top Gene Ontology (GO) biological process analysis and representative neurogenesis- and synaptic plasticity-related DEGs associated with OTT-induced transcriptional changes. **(n,o)** Integrated snRNA-seq analysis of saline-, young female plasma (YFP)-, sham- and OTT-treated hippocampi. **(p)** Overlap of parenchymal DEGs between YFP versus saline and OTT versus sham, showing conserved unidirectionally regulated DEGs. **(q–s)** Cell type-specific distribution and correlation of YFP- and OTT-regulated DEGs and the conserved signature. **(t,u)** GO biological process analysis and representative neurogenesis- and synaptic plasticity-related DEGs associated with the conserved transcriptional signature. Data are shown as mean ± s.e.m. Statistical analyses were performed using one-sample t-tests versus 0 (b) or 50% (c) (within-group comparisons), unpaired t-tests with Welch’s correction (b, f, between-group comparisons) and two-way ANOVA followed by Tukey’s multiple comparisons test (d). *P < 0.05, **P < 0.01, ***P < 0.001.

**Figure 3.**
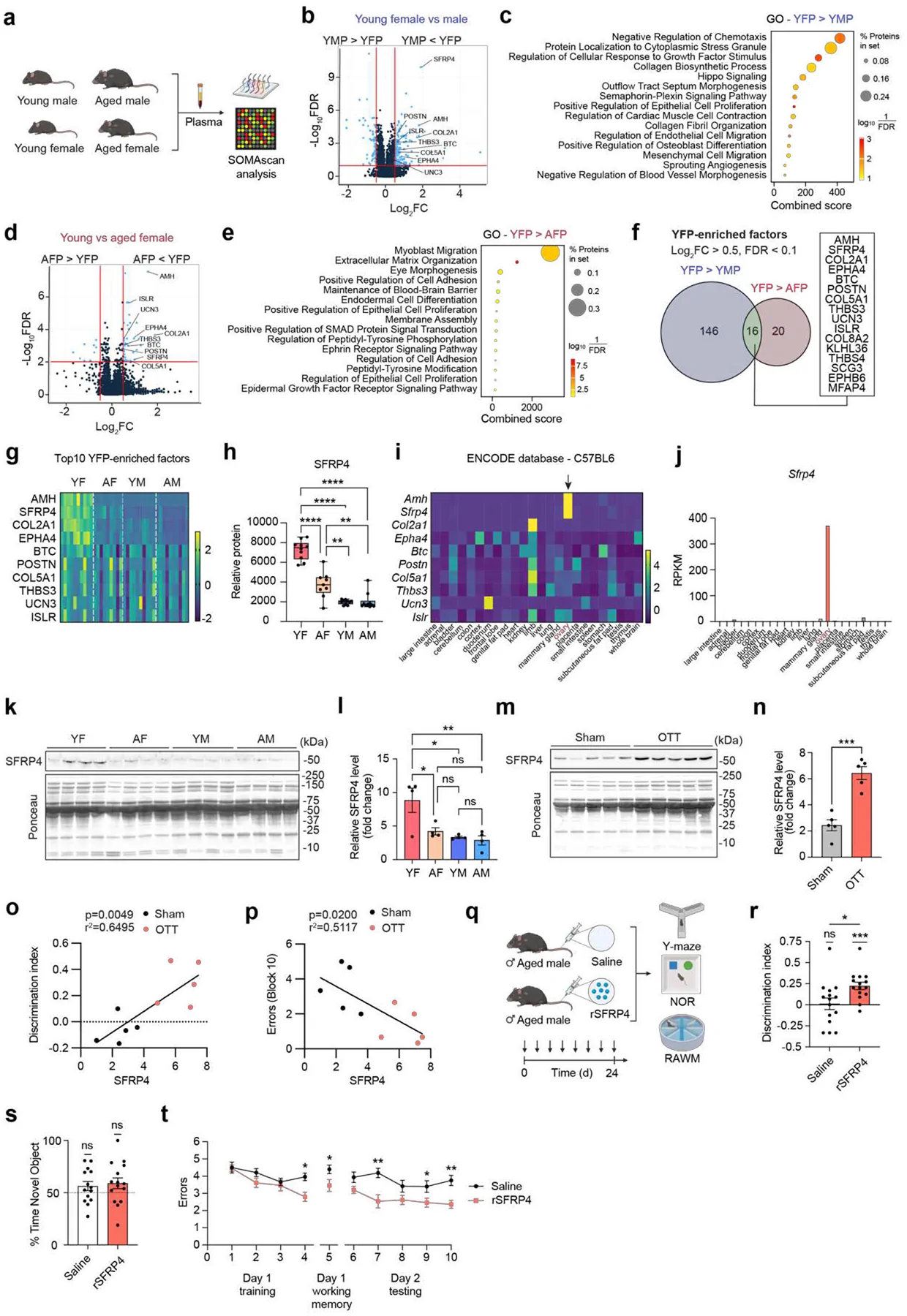
SFRP4 is an ovary-enriched factor that enhances cognitive performance in aged male mice. **(a)** Plasma from young male (YM; n = 10), young female (YF; n = 10), aged male (AM; n = 10), and aged female (AF; n = 9) mice was analyzed by SomaScan. **(b,c)** Volcano plot and Gene Ontology (GO) analysis of plasma differentially expressed proteins (DEPs) in YFP versus YMP. **(d,e)** Volcano plot and GO analysis of plasma DEPs in YFP versus AFP. **(f)** Venn diagram showing proteins shared between YFP versus YMP and YFP versus AFP, identifying 16 YFP-enriched proteins. **(g,h)** Top plasma DEPs and SFRP4 levels. **(i,j)** Tissue expression profiles of the top plasma DEPs and Sfrp4 based on ENCODE transcriptomic datasets. **(k,l)** Representative western blots, Ponceau S stains, and quantification of plasma SFRP4 in young and aged mice (n = 4 per group). **(m,n)** Representative western blots, Ponceau S stains, and quantification of plasma SFRP4 in sham and OTT mice (n = 5 per group). **(o,p)** Correlation of plasma SFRP4 levels with Y-maze performance and final-block RAWM errors. **(q)** Experimental design for repeated intravenous administration of recombinant mouse SFRP4 to aged male mice. **(r)** Spatial working memory was assessed by the Y-maze (n = 14–15 mice per group). **(s)** Object recognition memory was assessed by the novel object recognition (NOR) test (n = 14–15 mice per group). **(t)** Hippocampal-dependent spatial learning and memory were assessed by the radial arm water maze (RAWM) (n = 13–16 mice per group). Data are shown as mean ± s.e.m., unless otherwise indicated. Panel h is presented as a box-and-whisker plot (median, interquartile range, and min–max). Statistical analyses were performed using one-sample t-tests versus 0 (r) or 50% (s) (within-group comparisons), unpaired t-tests with Welch’s correction (n, r, between-group comparisons), one-way ANOVA followed by Tukey’s multiple comparisons test (h, l) and two-way ANOVA followed by Tukey’s multiple comparisons test (t).*P < 0.05, **P < 0.01, ***P < 0.001.

**Figure 4.**
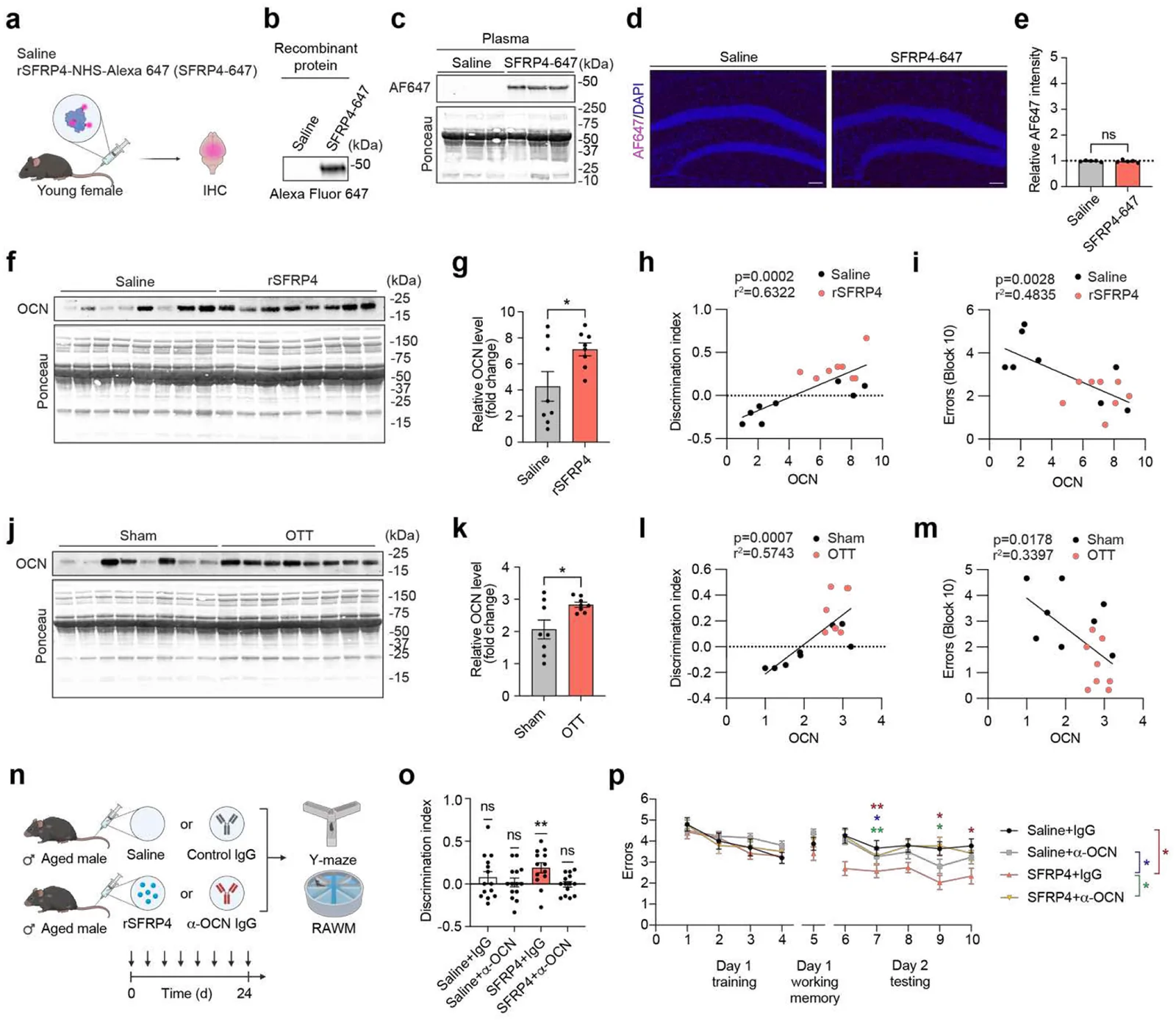
Osteocalcin mediates the cognitive effects of systemic SFRP4 in aged male mice. **(a)** Assessment of the permeability of Alexa Fluor-labelled SFRP4 (SFRP4–647) across the blood–brain barrier following intravenous injection in young (4-month-old) mice. **(b)** Western blots detecting 647-nm fluorescence in Alexa Fluor-conjugated SFRP4. **(c)** Western blots, corresponding Ponceau S stains, in plasma from saline- and SFRP4–647-treated aged mice (n = 3 per group). **(d,e)** Representative images and quantification of SFRP4–488 permeability across the blood–brain barrier (n = 5 mice per group; scale bar, 200 μm). The dotted line in **(e)** indicates background fluorescence (relative intensity = 1). **(f,g)** Representative western blots, corresponding Ponceau S stains, and quantification of plasma osteocalcin (OCN) in saline- and SFRP4-treated aged mice (n = 8 per group). **(h,i)** Correlation of plasma OCN levels with Y-maze performance and final-block radial arm water maze (RAWM) errors. **(j,k)** Representative western blots, corresponding Ponceau S stains, and quantification of plasma OCN in sham and OTT mice (n = 8 per group). **(l,m)** Correlation of plasma OCN levels with Y-maze performance and final-block RAWM errors. **(n)** Experimental design for repeated intravenous administration of recombinant mouse SFRP4 and an anti-OCN antibody to aged male mice. **(o)** Spatial working memory was assessed by the Y-maze (n = 13–14 mice per group). **(p)** Hippocampal-dependent spatial learning and memory were assessed by the radial arm water maze (RAWM) (n = 13–14 mice per group). Data are shown as mean ± s.e.m. Statistical analyses were performed using unpaired t-tests with Welch’s correction (e,g,k), one-sample t-tests versus 0 (o) and one-way ANOVA followed by Tukey’s multiple comparisons test (p). *P < 0.05, **P < 0.01.

## Data Availability

snRNA-seq data have been deposited in the Gene Expression Omnibus (GEO) under accession number GSE326930 and will be made publicly available upon publication. Processed SomaScan proteomeic data are also provided in Data S1.
